# Exploring the Isoprenoid Biosynthesis Pathway’s Role in Oncogenic Viruses

**DOI:** 10.3390/v18060637

**Published:** 2026-05-31

**Authors:** Louise N. Blaha, Jeffrey D. Neighbors, Richa Sandeep, Akhila Kondaka, Raymond J. Hohl

**Affiliations:** 1 Penn State Cancer Institute, Hershey, PA 17033, USA; lblaha@pennstatehealth.psu.edu (L.N.B.); jneighbors@pennstatehealth.psu.edu (J.D.N.); 2 Department of Molecular and Precision Medicine, Penn State College of Medicine, Hershey, PA 17033, USA; 3 Department of Medicine, Penn State College of Medicine, Hershey, PA 17033, USA; rsandeep@pennstatehealth.psu.edu (R.S.); akondaka@pennstatehealth.psu.edu (A.K.)

**Keywords:** oncovirus, isoprenoids, statin, prenylation, HMGCR, mevalonate, farnesyl pyrophosphate, geranylgeranyl pyrophosphate, cholesterol, microRNA

## Abstract

Oncogenic viruses, which are causative for some cancers, are typically acquired in youth and suppressed until older age. These malignancies account for over 10% of the cancer burden and often reprogram cellular metabolic pathways to promote their own survival and proliferation, often targeting lipogenic pathways to increase bioavailability of cellular products. The isoprenoid biosynthesis pathway (IBP) is an important lipogenic pathway that has been explored extensively for its dysregulation contributing to carcinogenesis, with notable discussion of statin therapy as a means of perturbing neoplasia. To our knowledge, we are the first group to provide a comprehensive discussion of the seven known oncogenic viruses and their reliance upon the IBP to promote tumorigenesis. As knowledge on this topic is limited, we aim to draw attention to a neglected area of the oncogenic research space, while also highlighting the use of inhibitors of the IBP as potential avenues for novel treatment options.

## 1. Introduction

Oncovirus-induced cancers account for approximately 12% of the total cancer burden [1]. Viruses are obligate parasites that depend on host cells for survival, often manipulating the metabolic environment and biochemical pathways of the organism they are harboring to promote their own survival. A consistent feature of viral oncogenesis is metabolic reprogramming, particularly of lipid and fatty acid metabolism, which supports virion production through increased synthesis of lipid biosynthetic precursors. These precursors create the products needed for cellular membranes and signaling molecules, thereby promoting cell proliferation [2]. The Isoprenoid Biosynthesis Pathway (IBP), also known as the Mevalonate pathway, is a central metabolic pathway responsible for the production of isoprenoids, organic compounds that regulate critical cellular functions including cell signaling, gene expression, membrane integrity, and sterol synthesis [3]. This pathway is best known for cholesterol synthesis, which supports downstream production of steroid hormones, vitamin D, bile acids, and lipoproteins essential for cell survival [4]. In addition, the IBP generates key intermediates, including farnesyl, pyrophosphate (FPP), and geranylgeranyl pyrophosphate (GGPP), which regulate signal transduction and homeostasis via posttranslational modifications in which farnesyl or geranylgeranyl groups are covalently added to signaling proteins [5]. These intermediates are particularly relevant to oncogenesis, as they modulate signaling proteins, such as Ras and Rho.

The IBP has emerged as a therapeutic target in cancer, supported by studies of isoprenoid inhibitors such as statins and bisphosphonates. The pathway receives ongoing attention due to the repurposing of statins as a form of cancer treatment, which work to competitively inhibit the enzyme HMG-CoA reductase (HMGCR), responsible for carrying out the rate-limiting step of the IBP [6]. Although statins are first-line therapy for hypercholesterolemia, they have also demonstrated anti-tumor effects, including decreased proliferation and increased cytotoxicity in cancer cell lines [7]. Inhibition of mevalonate production or loss of HMGCR expression leads to growth arrest or cell death, highlighting the pathway’s role in cellular viability [4]. Emerging evidence suggests that multiple oncoviruses exploit the IBP, particularly through upregulation of HMGCR activity and increased dependence on downstream isoprenoid intermediates, to support viral persistence and malignant transformation. This highlights a convergent metabolic vulnerability across virally driven cancers.

Currently, there are seven known oncoviruses that are recognized in the literature: Epstein–Barr Virus (EBV), Hepatitis B (HBV), Hepatitis C (HCV), Human Papilloma Virus (HPV), Kaposi Sarcoma associated Herpes Virus (KSHV) (also known as Human Herpes Virus 8), Human T cell Lymphotropic Virus (HTLV) and Merkel Cell Carcinoma (MCPyV). Human Immunodeficiency Virus (HIV, formerly HTLV-3) is also associated with malignancy, though it is typically excluded from this group due to being in the Human Lymphotropic family. In this review, we synthesize current evidence on the interaction between oncoviruses and the IBP, with a focus on shared metabolic mechanisms and their implications for targeted therapeutic strategies.

## 2. The Isoprenoid Biosynthesis Pathway

The IBP is an anabolic biosynthetic pathway, in which a series of enzymatic reactions converts acetyl-CoA into several downstream isoprenoid products (IPP, FPP, GGPP), sterols (cholesterol, lanosterol), and polyisoprenoids (dolichol) [8]. Isoprenoid compounds comprise two or more five-carbon units termed ‘isoprenes’ (C5H8), which possess an incredible diversity of functions due to variation in molecular structure [9].

**Figure 1:**
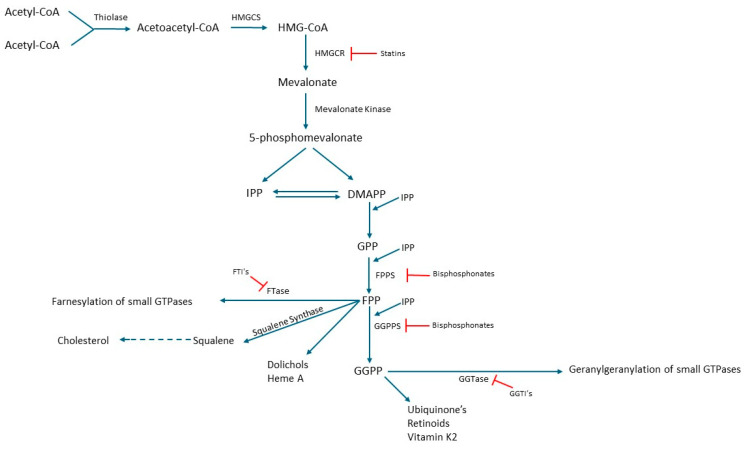
Schematic showing the steps of the IBP. Red arrows indicate inhibitory action, opposing doublet arrows represent interconversion and dashed arrows represent several reactions that have been excluded from the schematic for simplicity.

As shown in Figure 1, the IBP begins with the condensation reaction of acetyl-CoA and acetoacetyl-CoA to synthesize 3-hydroxy-methyl-glutaryl-CoA (HMG-CoA), which is carried out by the enzyme HMG-CoA synthase (HMGCS) [10]. Acetyl-CoA is typically derived as a product of the oxidative decarboxylation of pyruvate, which is delivered as the starting product of the IBP. Additionally, Acetyl-CoA can be fed into the citric acid cycle for energy production or used as a metabolite in fatty acid synthesis [11]. HMG-CoA is converted to mevalonate in the committed step of the IBP via HMG-CoA reductase (HMGCR) and NADPH. Mevalonate will undergo further transformation via mevalonate kinase to yield 5-phosphomevalonate. 5- phosphomevalonate will give rise to isopentenyl (IPP)—a five-carbon molecule that can undergo isomerization to form dimethylallyl pyrophosphate (DMAPP). A subsequent condensation reaction with IPP and DMAPP results in the ten-carbon molecule geranyl pyrophosphate (GPP). GPP is then elongated via the addition of an IPP group to form the fifteen-carbon molecule farnesyl pyrophosphate (FPP) in a reaction catalyzed by FPP synthase (FPPS). A subsequent IPP molecule will be added to FPP to form the 20-carbon molecule geranylgeranyl pyrophosphate (GGPP) in a condensation reaction catalyzed by GGPP synthase (GGPPS). Nitrogenous bisphosphonates (e.g., zoledronic acid) are a class of drugs that inhibit FPPS, reducing FPP production and demonstrating potential as IBP-targeted therapies beyond their traditional use in bone resorption disorders [12]. In fact, some studies highlight that combination treatment of bisphosphonates and statins is able to combat the off-target effects of using each drug independently, while maintaining decreased cholesterol levels [13].

Because isoprenoids are subject to a variety of chemical modifications, many longer and more complex molecules are produced as end products of IBP. For example, FPP is a precursor to sterols, dolichols, and Heme A., while GGPP contributes to the synthesis of ubiquinone, retinoids, and vitamin K2 [3,10]. Of importance is FPP and GGPP’s ability to act as substrates in key post-translational modification processes known as protein prenylation, in which farnesyl or geranylgeranyl moieties are added to signaling proteins to assist in membrane anchoring and localization [14]. Farnesyl transferases (FTases) and geranylgeranyl transferases (GGTases) are enzymes that catalyze protein prenylation, particularly of the Ras superfamily of small GTPases.

The Ras superfamily comprises over 150 proteins divided into five subfamilies (Ras, Rho, Rab, Arf, and Ran), each regulating diverse cellular processes. Post-translational modifications such as prenylation control the subcellular location of Ras proteins and enable interactions with a multitude of signaling pathways [15]. Tumor pathogenesis has been linked to the activity of prenylated GTPases; thus, inhibitors of GTPase prenylation are being explored for their anticarcinogenic properties [16]. Viruses have been proposed to accelerate carcinogenic processes, thus may also act to dysregulate the IBP to promote viral infection and propel viral oncogenesis [17].

## 3. Cholesterol Function

Cholesterol is an essential lipid that has functions in membrane rigidity, cellular organization, and signal transduction and serves as a precursor molecule to sterol-derived hormones such as vitamin D and bile acids [7]. In de novo cholesterol synthesis, FPP acts as a precursor, as two FPP molecules condense to form presqualene synthase, which is further processed into squalene and then cholesterol [13].

Although cholesterol represents a major output of the IBP, it can also be obtained exogenously through plasma lipoproteins (high-density and low-density), which transport cholesterol obtained from dietary intake or from preexisting stores to the appropriate cellular locations for use, storage, or disposal [18]. A balance between both exogenous and endogenous cholesterol levels must be obtained to maintain appropriate concentrations of sterols and isoprenoid constituents, so as not to disturb critical cellular functions. For example, increased low-density lipoprotein levels inhibit HMG-CoA synthase and reductase activity by up to 90%, thereby decreasing endogenous production of cholesterol until cholesterol stores are depleted [4].

Viruses depend upon host cholesterol metabolism to regulate viral entry and viral morphogenesis. Cholesterol-rich membrane domains facilitate viral entry, while host-derived lipids are required to form viral envelopes [19]. Given the central role of the IBP in cholesterol production, many oncoviruses exploit this pathway to increase lipid availability, supporting both viral propagation and oncogenic signaling. This reflects a broader trend in which viral-driven cancers converge on IBP-mediated lipid regulation as a shared metabolic dependency.

## 4. Regulation of the IBP

The IBP occurs in the cytosol and at the Endoplasmic Reticulum (ER) and must be highly regulated to control sterol and isoprenoid biosynthesis. Regulation occurs primarily through sterol-dependent negative feedback mechanisms, where low concentrations of IBP end products stimulate translocation of regulatory proteins to promote IBP activity and restore homeostasis.

Sterol regulatory element binding proteins (SREBPs) are a family of membrane-bound transcription factors that form complexes with SREBP cleavage activating protein (SCAP) in response to low sterol levels. SREBP-SCAP complexes are transported from the ER to the Golgi apparatus for processing via S1P and S2P proteases, along with subsequent translocation of SREBP (particularly SREBP-2) to the nucleus to upregulate genes involved in isoprenoid biosynthesis, including HMG-CoA synthase (HMGCS), HMG-CoA reductase (HMGCR), and mevalonate kinase [18,20]. On the other hand, when cholesterol levels rise above 5%, cholesterol particles bind to SCAP and prevent SREBP translocation to the Golgi. This occurs through SREBP-SCAP binding to ER resident protein insulin-induced gene 1, which together block the movement of the SREBP- SCAP complex away from the ER, thus preventing activation in IBP genes [21].

This regulatory axis represents a critical control point exploited by oncoviruses. Several viruses modulate SREBP signaling or disrupt sterol-mediated feedback to sustain IBP activity, ensuring a continuous supply of lipids and isoprenoid intermediates required for viral replication and oncogenic signaling. This highlights SREBP–SCAP regulation as a shared mechanistic link between viral infection and metabolic reprogramming in cancer.

Over the next sections, the role of the IBP in sustaining tumorigenic behavior in each oncovirus will be discussed, with a summary of these findings presented in Table 1.

## 5. Epstein–Barr Virus

Epstein–Barr Virus (EBV) is a DNA virus in the Herpesviridae family associated with malignancies such as Burkitt lymphoma, Hodgkin lymphoma, gastric carcinoma, and nasopharyngeal carcinoma [59]. EBV was the first human tumor virus to be discovered in 1964 [60]. Subsequent studies confirmed its oncogenic potential, and it is now classified as a Group 1 carcinogen [61].

EBV is a ubiquitous virus, affecting roughly 90% of the population, typically starting in childhood, and establishes latency in B lymphocytes and epithelial cells. During latency, EBV remodels host biosynthetic pathways to drive transformation of quiescent B cells into proliferative states [22,62]. EBV carcinogenesis relies upon the activities of viral oncoproteins EBV nuclear antigens (EBNA1/2/3A/3B,3C and LP) and latent membrane proteins 1 /2 (LMP1/LMP2), which regulate viral replication and latency, as well as cell proliferation, differentiation, and apoptosis [59].

EBV has been studied for its ability to remodel host signal transduction, particularly regarding how remodeling of metabolic pathways supports viral infection and leads to cancer development [23]. Wang et al. were able to demonstrate through proteomic dataset analysis of major biosynthetic pathways that EBV induces fatty acid and cholesterol synthesis pathways to promote B-cell growth. The researchers were able to narrow down these effects to the IBP, in which GGPP activity was most highly responsible for EBV B cell outgrowth. Within two days post-infection, HMGCS1 and HMGCR were among the most upregulated enzymes, highlighting early activation of the IBP. Surprisingly, addback experiments of cholesterol precursor squalene after statin treatment failed to rescue cells, suggesting that cholesterol itself is not the primary driver of early EBV-mediated transformation. In contrast, GGPP supplementation restored EBV-driven B-cell transformation, implicating isoprenoid intermediates rather than cholesterol as critical mediators. This effect was linked to GGPP-dependent activation of Rab GTPases. Out of the 24 Rab proteins detected to have upregulated activity in lymphoblastoid cell lines, GGPPs role in Rab13’s activation was identified for its ability to act as a chaperone for oncoprotein LMP1 and LMP2A trafficking [22]. This finding highlights the IBPs and, specifically, GGPPs’ role in promoting B-cell transformation by EBV.

These researchers also explored the regulation of the pathway by SREBP and SCAP to determine their roles in EBV. SPEBP2 was found to be highly expressed in lymphoblastoid cell lines, and the EBNA2 oncoprotein occupies the SREBF2 promoter region in these cells. Through the use of agents that block SCAP translocation from the ER to the Golgi, SCAP was determined to play a role in EBV-induced B-cell growth as measured by mRNA abundance of *ACLY* and *SCD* (genes targeted by SREBP) and increased cell death. EBNA2, RBP-Jk, MYC, and MAX were all found to occupy *HMGCR* and *SREBP2* promoter regions, which, given their role in oncogenesis, point to the importance of the IBPs in control of EBV infection. IBP-associated genes that are dysregulated in EBV are denoted in Table 1.

In a study that analyzed the effects of simvastatin on the growth of EBV-transformed lymphoblastoid cell lines, it was shown that 2 μM doses of simvastatin were high enough to induce apoptosis and inhibit clump formation of lymphoblastoid cell lines. This effect is partly mediated through disruption of leukocyte function antigen-1 (LFA-1) interactions within lipid rafts, leading to downregulation of NF-κB signaling downstream of LMP1 and subsequent apoptosis [24]. Others have shown that simvastatin treatment impairs EBV-induced B-cell outgrowth, as well as highlighting other statins, such as atorvastatin, that similarly impair EBV-driven B-cell growth and survival [22]. These findings are summarized in Table 2.

While the current literature surrounding IBP dysregulation in EBV focuses on B-cell transformation, emerging evidence suggests that similar metabolic reprogramming may contribute to EBV-associated malignancies such as nasopharyngeal carcinoma and gastric carcinoma. One study demonstrated that EBV-encoded RNAs (EBERs) in nasopharyngeal carcinoma upregulate lipid metabolism processes, as shown through gene ontology analysis. Genes coding for LDL receptors and fatty acid synthase were shown to be upregulated, thus leading to the conclusion that deregulated lipid metabolism is partially responsible for alterations in cellular signaling and cell proliferation that lend themselves to tumorigenesis [70]. Additionally, other nasopharyngeal carcinoma research has demonstrated a link between EBV-associated proteins such as LMP1 and EBNA2 and increased lipid synthesis, such as SREBP activation [22,25]. Because prenylated signaling proteins are critical regulators of viral latency and innate immune responses, it is possible that increased IBP flux may support epithelial oncogenesis via a modulation of lytic reactivation and innate immune response pathways. For instance, BRLF1 and BZLF1 have been shown to regulate fatty acid synthesis during lytic activation, while altered cholesterol metabolism has been implicated in the regulation of antiviral responses; thus, it is possible that IBP activity may influence EBV transcription factors responsible for lytic reactivation [63,71]. Increased IBP activity may also contribute to immune escape mechanisms, as cholesterol metabolism and prenylation can influence membrane signaling and innate immune response pathways such as cGAS-STING [64,65]. IBP hyperactivation may additionally contribute to immune evasion during EBV-associated carcinogenesis; however, this proposed relationship remains poorly understood.

The LMP1 oncoprotein has been explored for its ability to promote EBV-driven metastasis in the context of key cancer regulators, exosomes [22]. Exosomes are extracellular membrane vesicles that function to transport molecules for intercellular signaling purposes. LMP1 is loaded into exosomes to later be secreted during cancer invasion and metastasis processes. Farnesylation at the C-terminal site of the molecule ubiquitin C-terminal hydrolase-L1 (UCH-L1) is a critical lipid modification that allows for LMP1 exosome loading, thus promoting neoplasia. Through the use of small molecule inhibitors (farnesyltransferase inhibitors) that target farnesylation of UCH-L1, researchers highlight farnesylation as a potential target against cancer cell invasion induced by EBV. This is demonstrated through FTI-277’s ability to tamper with cell motility and migration as well as decrease anchorage-independent growth of EBV-positive cells [26].

Data on microRNA-mediated regulation of the IBP in EBV remains limited, representing an area for future investigation.

## 6. Hepatitis B (HBV)

Hepatitis B virus (HBV) is a small, enveloped, partially double-stranded DNA virus of the Hepadnaviridae family that shares several replication characteristics with retroviruses [72,73]. Although initially discovered in the 1960s, its definitive causative role in hepatocellular carcinoma (HCC) was established in the 1980s [74]. HCC, typically arising from persistent HBV or HCV infection progressing through chronic liver disease and cirrhosis, remained the third leading cause of cancer deaths in 2023 despite widespread vaccination [75]. Chronic Hepatitis B infection (CHB) is usually underlined by high plasma levels of HBV-associated viral antigen (HBsAg), which circulates in noninfectious vesicles that comprise several lipid types. The secretion of HbsAg is a critical marker of infection, often signaling a suppressed host immune response and an inability to clear the virus [27].

HBV has been studied extensively for its ability to remodel lipid biosynthesis, and the mechanisms by which altered lipid metabolism occurs have been explored in HBV cell lines and mouse models. Critical cholesterol and IBP-regulating genes such as *SREBP2* and HMGCR have been shown to be enhanced compared to controls in HBV mouse models [28]. In HepG2 lines, upregulated mRNA levels of *HMGCR* and *LDLR* promote significant cholesterol accumulation, while metabolomic profiling of HBV-infected mice shows a concomitant increase in proteins associated with lipogenesis and fatty acid oxidation [29,30]. Notably, an upregulation of 3-hydroxy-3-methylglutarate in some models may represent a negative feedback response attempting to diminish IBP activation in the face of virus-induced cholesterol surges [27].

A 2022 meta-analysis reviewed the relationship between statin users and risk of developing HBV and HCV-induced HCC and found that statins significantly decreased this risk, particularly among HBV/HCV cirrhotic patients [31]. Mechanistic studies using Lovastatin in Hep3B and HepG2 lines demonstrated a dose-dependent inhibition of HBsAg secretion without altering cellular proliferation or viral transcription, suggesting that statins specifically target the cholesterol biosynthesis pathways required for viral envelope assembly [32]. A more recent group attempted to recreate the results described above using lovastatin to reduce HBsAg levels; however, in HepG2-NTCP cells, they failed to demonstrate lovastatin’s inhibitory activity upon HBsAg secretion, attributing it to the absence of serum-starved media protocols [27]. Simvastatin has also been reviewed for its anti-HBV effects in the HepG2.2.15 cell line. Bader and Korba demonstrated that simvastatin elicits strong anti-viral effects upon in vitro HBV activity and displays synergistic antiviral activity when combined with known anti-HBV nucleoside analogs (lamivudine, adefovir, tenofovir, and entecavir [33]. Simvastatin has been further analyzed in a combination drug therapy study that explored the anticancer effects of simvastatin combined with NS398 (a selective cyclooxygenase-2 inhibitor) in Hep3B and Huh-7 lines. Combined, these two agents show high proapoptotic and antiproliferative effects upon each cell line, as shown by reduction in NF-kB, Akt, procaspase 3, and cyclin D1 measurement [34].

MicroRNAs (miRNAs) have been studied in the context of HBV, as they have displayed perturbation of viral replication processes and act as post-transcriptional regulators of genes involved in maintaining cholesterol homeostasis. In human hepatoma cells, miR-21 and miR-27 act to regulate the key IBP enzyme HMGCR. Specifically, miR-27 expression is frequently elevated in CHB, where it appears to mediate cholesterol production through the IBP, highlighting it as a potential therapeutic target for disrupting HBV-induced lipid remodeling [19,76].

## 7. Hepatitis C (HCV)

Hepatitis C virus (HCV) is a single-stranded positive-sense linear RNA virus of the Flaviviridae family that infects roughly 180 million individuals worldwide [77]. HCV and HBV together contribute to the leading cause of HCC, which in 2020 led to 830,000 deaths and 906,000 newly diagnosed cases globally [75]. The first major milestone in HCV diagnosis came about in the late 1980s in the form of an HCV antibody detection kit, which contributed to the initiation of the now-established relationship between HCV and HCC [74].

Not dissimilar to HBV, successful HCV viral life cycles depend upon host cell cholesterol biosynthesis for the construction of new virions, viral replication, and viral entry into hepatocytes [78]. Both lipophilic and hydrophilic statins have been shown to perturb HCC risk through their inhibitory action upon HMGCR, with lipophilic statins having particularly potent effects. Atorvastatin, a lipophilic statin, was shown to decrease the PLS score (a clinical liver prognostic measure that predicts HCC risk) in Huh7.5.1 cell lines by downregulating the unfolded protein response and interferon signaling.

Atorvastatin further disrupts oncogenesis by targeting YAP (Yes-associated protein), a key effector of the Hippo signaling pathway that drives cell proliferation [35]. In HCV-infected controls, YAP-regulated genes are typically upregulated; however, atorvastatin treatment increases the phosphorylation of YAP at the S127 and S397 sites. This modification prevents YAP from translocating into the nucleus, rendering it unable to promote the expression of pro-growth target genes. Because the addition of GGPP successfully rescued YAP activity in these models, researchers concluded that HCV-induced YAP activation is specifically mediated through the mevalonate pathway [35,36].

In research exploring genotype 1 HCV replicons, HCV RNA replication is seen to rely upon the mevalonate pathway for cholesterol synthesis, as the byproduct geranylgeranyl is used as a lipid substrate by HCV-expressing cells. Covalent attachment of a geranylgeranyl group to the COOH-terminus of the F-box protein FBL2 allows for FBL2 association with NS5A (HCV replication complex non-structural protein), a critical step required for HCV RNA replication. This finding was supported by experiments that demonstrated decreased HCV RNA replication using HMGCR inhibitor lovastatin and then recovered RNA replication with the addition of geranylgeraniol but not cholesterol or farnesol [37]. To determine the importance of GGTase 1 and FTase upon HCV replication, Huh7 cells were treated with increasing doses of both GGTase I inhibitor GGTI-298 and FTase inhibitor FPTI-III. GGTI-298 but not FPTI-III caused a dose-dependent decrease in HCV RNA. GGTI-298 also caused a dispersion of the NS5A protein, leading to the conclusion that GGTase I is critical for the proper functioning of the HCV replication machinery [38].

Lovastatin has also been studied in HCV-replicating R-1 cells, in which 10 μM doses for 48 h decreased viral genome abundance by up to 70%. Monascus orange pigment (MOP) amino acid derivatives have been evaluated for their ability to inhibit HCV replication through modulation of the IBP downstream of mevalonate. MOP compounds display antiviral activity through their inhibition of the mevalonate pathway and subsequent interference with interactions between FBL2, NS5A, and HCV. To determine this, Huh7 R-1 cells were treated with mevalonate in the presence and absence of various MOP amino acid derivatives, then tested for their HCV RNA levels. Mevalonate alone resulted in an increase in HCV RNA, while MOP conditions reduced HCV RNA and cholesterol levels, indicating that MOP derivatives inhibit HCV replication via interference with the IBP [39]. Other naturally derived compounds, such as Curcumin, Cinnamon oil, Vitellaria paradoxa, and Berberis, have also been shown to reduce or inhibit cholesterol production in HCV through their action as HMGCR inhibitors [40].

HCV utilizes Rab GTPases, such as Rab5A, Rab7, and Rab18, to regulate the intracellular trafficking required for viral infection, including vesicle budding and uncoating. For these “molecular switches” to function at host membranes, they must first undergo geranylgeranylation via the isoprenoid biosynthetic pathway (IBP). While targeting this process with lipid transferase inhibitors offers a potential therapeutic avenue, the clinical utility is currently limited by the systemic toxicity and dangerous off-target effects associated with blocking isoprenoid production [41]. Because HCV persistence post-antiviral therapy has been linked to HCV RNA presence in extrahepatic tissue, peripheral blood mononuclear cells have been explored for their modulation via the IBP. After antiviral treatment, peripheral blood mononuclear cells were analyzed for the presence of HMGCR and GGPPS, then compared with simultaneous HCV RNA expression. As peripheral blood mononuclear cells were discovered to have increased expression of GGPPS, it was hypothesized that HCV RNA alters the IBP as a means to alter the metabolic balance between GGPP and cholesterol [42].

**Figure 2:**
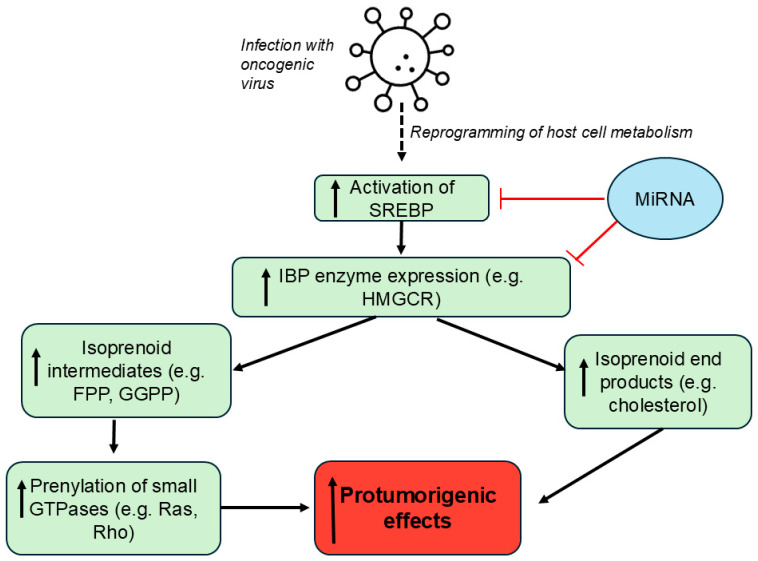
Proposed mechanism between viral infection and IBP dysregulation for oncoviruses. Viral activation of SREBP and increased IBP activity drive protumorigenic signaling. Red arrows indicate inhibitory action, upward pointing arrows are indicative of increased activity and dashed arrows indicate several unmarked steps. Virus icon from Microsoft PowerPoint.

MicroRNAs serve as crucial post-transcriptional modulators in viral hepatitis, targeting the isoprenoid biosynthetic pathway (IBP) to inhibit viral replication and counteract oncogenic changes [19]. As denoted in Figure 2, this research indicates that miRNAs typically repress the activity of *SREBP* and *HMGCR*, to exert control over the IBP, potentially acting to inhibit changes induced by viral oncogenesis. While miR-122 is highly expressed in the liver and correlates with HCV RNA abundance, experimental inhibition suggests its influence on viral accumulation may be independent of its effects on HMGCR levels [79]. In contrast, miR-21 and miR-27 are frequently dysregulated in chronic hepatitis C and HCC, where they directly regulate lipid homeostasis. Overexpression in Huh7 cells significantly inhibits cholesterol production by 70% and 30%, respectively, with miR-27 specifically binding to the 3′ UTR of *HMGCR* to repress synthesis [76]. The host’s miRNA-driven defenses are actively contested by viral proteins. The HCV core protein represses miR-185-5p to increase SREBP2, cholesterol levels, and HMGCR expression [80]. Additionally, the HCV Nonstructural 4b protein promotes lipid production through the AKT signaling pathway and synergistic interactions with the core protein to perturb SREBP-1 activity [81]. Together, these interactions highlight a complex metabolic tug-of-war between host regulatory RNAs and viral proteins.

## 8. Human Papillomavirus (HPV)

Human papillomaviruses (HPV) are double-stranded DNA viruses of the Papovaviridae family [82]. While mostly attributed to cervical cancer, HPV infection also impacts the genital tract, oral, oropharyngeal (head and neck squamous cell carcinoma), and respiratory regions [83]. Although infectious skin and genital warts have been documented for centuries, HPV’s role as an oncovirus was not hypothesized until the 1970s. To date, there are over 100 distinct forms of HPV, and estimates reveal that more than 85% of sexually active people catch HPV [84].

Statins show potential as antitumorigenic agents in HPV, primarily through dose-dependent antiproliferative effects. In one study where atorvastatin, fluvastatin, and simvastatin were tested across HPV positive and negative cell lines, statin treatment resulted in a decrease in cell viability across lines, most notably in the viral-negative ViBo line. Cell death post statin treatment was determined to be independent of cell cycle arrest but was instead observed to be due to necrosis and apoptosis, coupled with increased oxidative stress via reactive oxygen species (ROS) and nitrate production [43]. In addition, statins were observed to have a more prominent protective effect in cases of HPV positive HNSCC patients as compared to HPV negative, when analyzing the rate of disease-specific death and rate of disease recurrence [66]. However, a separate study also researching HPV negative HNSCC concluded that patients who used statins had increased overall survival and disease-specific survival outcomes as compared with controls [67].

Statins exhibit immunomodulatory effects in HPV-associated cancers. Specifically, statin use correlates with increased tumor-infiltrating lymphocytes (TILs) in HPV-positive head and neck squamous cell carcinoma samples, though no association has been found with systemic cytokines like IL-6 [68]. This suggests that a robust, virus-induced immune response may synergize with statins to improve clinical outcomes [68].

To elucidate an understanding of the most critical constituents of the IBP for pro-tumorigenic behavior, one group performed addbacks of mevalonate, FPP, and GGPP post statin treatment across three cervical cancer cell lines. While ViBo (HPV−) cells responded to addback treatment with a 100% rescue post statin treatment, both HPV+ cell lines responded with a meek 33% rescue, indicating an uneven reliance upon the IBP across viral positive and negative cell lines [43].

The cholesterol derivative 25-Hydroxycholesterol (25HC) exerts a dose-dependent antiviral effect by inhibiting the IBP enzymes FTase and GGTase. This inhibition disrupts the prenylation of Rho-family GTPases—including RhoA, Cdc42, Rac1, and Rap1—which are essential for HPV infection [44]. Furthermore, GGTase inhibition via GGTI-298 prevents the phosphorylation of LIMK1 and cofilin, proteins that regulate the actin cytoskeletal dynamics necessary for tumorigenic behavior. These findings underscore the critical role of GGPP in maintaining the structural machinery required for HPV infection and progression [44].

While no studies have established a baseline expression of IBP related genes associated with HPV infection, in a study that analyzed the cytotoxic effects of bacterial cyclodipeptides (CDPs) (small molecule exhibiting broad anticancer effects) upon HeLa cells, RNA sequencing revealed an upregulation of six genes collectively involved in the IBP and cholesterol pathways (*HMGS1*, *HMGCR*, *IDI1*, *MSMO1*, *TECR*, *LRRK2*) in response to the presence of the bacterial cyclodipeptides. mRNA transcript levels also matched these results, and cholesterol levels decreased in CDP-treated samples, suggesting that the IBP and downstream protein prenylation are key pathways for the proliferation of HeLa cells. When HeLa cell supernatant media were analyzed for metabolites, IBP intermediates were measured to have accumulated in the media, while cholesterol levels were diminished. This relationship mirrored the statin treatment condition, indicating that CDPs may inhibit the HMGCR enzyme. As a consequence, auto-synthesis of cholesterol and IBP intermediate accumulation occurs to support pro-tumorigenic behavior in HeLa cells [85].

Currently, there is no research regarding microRNA regulation of the IBP in HPV.

## 9. Kaposi Sarcoma Associated Herpes Virus (KSHV)

Kaposi sarcoma- associated herpes virus (KSHV), also known as human herpes virus 8 (HHV-8), is an enveloped double-stranded DNA virus that belongs to the Herpesviridae family [86]. The virus was discovered in 1994 as the infectious agent underlying Kaposi sarcoma (KS), a vascular tumor that affects several tissue types such as skin, mucosa, and viscera. KS does not arise from KSHV infection alone but is rather associated with acquired immune deficiency syndrome (AIDS) since the 1980s [87]. At present, very limited data exist surrounding the role of the IBP in KSHV oncogenesis, and there are no studies evaluating statin use and KSHV [88].

There is some evidence to support KSHV’s reliance upon the IBP to support pro-tumorigenic behavior. One group has discovered that KSHV encodes viral miRNAs that work in part to repress expression of multiple key enzymes of the IBP, including HMGCS1, HMGCR, and Farnesyl-diphosphate farnesyltransferase 1 (codes for squalene synthase) [45]. In their methodology, they compared transfection of HUVEC cells with viral miRNAs that target multiple genes in the IBP, to small interfering RNAs (siRNAs) which target *HMGCS1* only. Cholesterol levels were reduced by the miRNA transfection and not by the siRNA transfection, leading to the idea that the IBP’s complex nature requires repression of multiple genes to elicit a cellular effect. They explored this further as they compared a mutant KSHV condition, which lacked 10/12 of the miRNAs that the wild-type harbored, and observed decreased cholesterol levels in the wild-type compared with increased cholesterol levels in the mutant condition [46].

While research on the reliance of KSHV on the IBP is limited, there are a few studies that have explored the reliance of KSHV infection upon lipid rafts and droplets. Lipid rafts are critical structures that use cholesterol and other lipids to form microdomains, which function to compartmentalize signal transduction processes. They are also implicated in cancer since cancerous cells often have high amounts of lipid rafts compared to non-cancerous cells, as they rely upon these structures for pro-tumorigenic processes [89].

When HMVEC-d cells were treated with cholesterol sequestering agents mβCD and nystatin to disrupt lipid rafts, expression of viral genes ORF50 and ORF53 decreased. Lipid raft disruption also increased viral DNA internalization, disrupted microtubules, induced plasma membrane morphological changes, and affected a plethora of signaling processes. Such changes highlight the importance of lipid rafts for KSHV infection. Because lipid rafts rely upon cholesterol for their functionality, IBP may be a critical pathway in promoting KSHV viral infection, as it feeds a significant amount of cholesterol into cells [47].

Cholesterol is a major component of lipid droplets as it is stored in these structures in its cholesterol ester form [90]. In one study, cholesterol ester synthesis in HUVEC cells was observed to increase during the latent stage of KSHV viral infection, and when inhibited, impaired cell neo-tubule activity as measured by microtubule formation in HHV8-infected cells. Capillary microtubule formation is associated with neo-angiogenesis, which is a prerequisite to lesion formation associated with Kaposi’s angiosarcoma and is often heightened with HHV-8 infection. This property was thus measured to determine if cholesterol esterification played a role in promoting processes enhancing infection and subsequent malignancy [48]. Once again, KSHV’s perceived reliance upon cholesterol to carry out viral function suggests a hyperactivation of the IBP and demands an exploration of this pathway in the context of KSHV.

In later work, Serquiña et al. expanded upon their initial hypothesis to include the possibility that KSHV miRNAs were working to decrease cholesterol levels so that 25HC (derivative of cholesterol) production would be minimized and could not exert its antiviral effects. 25HC treatment of HUVEC cells decreased viral gene expression as measured by a decrease in levels of both viral replication and transcription activator (RTA) and latency-associated nuclear antigen [69]. As outlined in Table 3, two KSHV viral miRNAs were observed to repress cholesterol 25-hydroxylase (CH25H) (an enzyme that converts cholesterol to 25HC), and overall levels of CH25H were increased during initial infection periods and reduced after a period of latency [46]. RNA sequencing analysis revealed that 25HC induced gene expression of interferon-stimulated genes and inflammatory cytokines (IL-8 and IL-1α), indicating that 25HC may be acting through the innate immune system as a defense mechanism [69].

## 10. Human T Cell Lymphotropic Virus (HTLV-I)

Human T cell Lymphotropic Virus (HTLV-I) is an RNA virus that was the first human virus of the Retroviridae family to be discovered in 1980. Although four distinct strains of HTLV-I exist (HTLV-I/II/III/IV), only HTLV-I has been associated with diseases such as Adult T cell Leukemia-Lymphoma (ATL), HTLV-I-Associated Myelopathy/Tropical Spastic Paraparesis, HTLV-I Associated Arthopathy, and Cutaneous T Cell Lymphoma [91]. HTLV-I primarily targets immune cells, and the main routes of infectious transmission are via sexual intercourse, breastfeeding, and organ transplant [92].

The IBP has been investigated in HTLV-I leukemia cell lines through the use of inhibitors such as statins, bisphosphonates, and transferase inhibitors. Statins, including lovastatin and simvastatin, reduce the viability of ATL cells in a dose-dependent manner, an effect significantly more prominent in HTLV-1-infected cells than in HTLV-1-negative CEM cells [49]. Addback experiments demonstrate that cell viability post-statin treatment can be rescued by FPP and GGPP, but not by squalene, indicating that the distal cholesterol pathway is less critical than prenylation intermediates. This is supported by the observation that statins inhibit the prenylation of Rab5B and Rac1 proteins [49]. Similarly, treatment with the bisphosphonate Incadronate leads to decreased cell viability, S-phase arrest, and caspase-dependent apoptosis specifically in HTLV-I-positive lines [50]. While both geranylgeraniol (GGOH) and farnesol (FOH) can reverse these effects, GGOH exhibits stronger restoration, and Incadronate treatment notably increases levels of unprenylated Rap1A in infected cells [50].

The importance of geranylgeranylation is further highlighted by studies using the transferase inhibitors GGTI-298 and FTI-277. GGTI-298 significantly reduces ATL cell viability and causes G2/M phase accumulation, whereas FTI-277 does not, suggesting a cellular preference for Rho GTPase-led signaling processes that require GGPP for activation [49,51]. Furthermore, GGTI-298 treatment inactivates the NF-kB pathway and inhibits HTLV-1 long terminal repeat transcription through a p53-independent mechanism [51]. These combined results indicate that GGPP is critical for cellular survival in HTLV-I-associated ATL, likely due to its role in activating Rho GTPase signaling [49,51].

HTLV-I viral proteins also directly interact with the IBP to control cellular processes. The accessory protein ${p13}^{II}$ targets mitochondria and interacts with FPPS, the enzyme converting IPP to FPP [52,53]. Inhibiting farnesylation in ${p13}^{II}$-expressing cells reduces apoptosis, suggesting that farnesylation may be essential for virus-induced survival [54]. Additionally, the viral proteins HBZ and Tax dysregulate the IBP to increase the pool of intermediates, particularly IPP, for use by T-cell receptors [93]. Despite these detailed metabolic interactions, the role of microRNA regulation of the IBP in HTLV-1 remains currently undescribed.

## 11. Merkel Cell Polyomavirus (MCPyV)

Merkel cell polyomavirus (MCPyV) is a small (5000 bp), circular, non-enveloped double-stranded DNA virus that belongs to the Polyomavirdae family. In 2008, MCPyV was sequenced from Merkel cell carcinoma (MCC) tumor samples and has since been confirmed to underlie most cases of MCC, making it the most recent addition to the oncovirus group. It is also the only human polyomavirus to be strongly associated with malignancy [94]. MCC is a rare but highly aggressive cutaneous neuroendocrine malignancy that primarily affects the elderly and immunocompromised population [55]. Although MCPyV is a relatively ubiquitous virus, the exact carcinogenic mechanisms underlying MCC are uncertain. To date, IBP has not been looked at with respect to MCC development; however, some adjacent topics have been investigated.

Increased IBP flux is implicated in Merkel cell carcinoma (MCC) pathogenesis, as demonstrated by the effects of the XPO1 inhibitor selinexor. Although primarily a nuclear export inhibitor, selinexor reduces the expression of SREBP1/2 and squalene synthase, key transcription factors that regulate lipogenesis and the IBP, thereby perturbing MCC cell growth [56]. Furthermore, genome sequencing has identified “gain of function” mutations in HRAS and PIK3CA, and experimental models confirm that MCC is highly sensitive to PI3K/AKT inhibition [57,58]. Given that the PI3K-AKT pathway is closely interconnected with the IBP, dysregulation of these metabolic intermediates likely influences MCC oncogenesis.

The role of the IBP in metastatic behavior is further suggested by its link to Rho signaling and viral antigens. The MCPyV small tumor antigen, essential for viral replication, has been shown to control filopodial formation and cellular motility through Rho GTPase signaling. Because Rho GTPases require prenylation for functional activation, the IBP likely serves as a critical driver of MCC motility [55]. Despite this, clinical evidence remains limited to correlational studies; while some data suggest a higher incidence of MCC among statin users, these studies are limited by small sample sizes and a lack of data regarding the viral status of patients [95,96]. Consequently, the precise role of the IBP in MCPyV-mediated tumorigenesis requires further investigation.

## 12. Conclusions

The goal of this review was to highlight the mechanisms by which oncoviruses dysregulate the IBP to accelerate carcinogenic behavior, and to draw similarities between shared features of each of the seven oncoviruses with respect to a key metabolic pathway. Although oncogenic viruses vary in their genome makeup and structure, as demonstrated in Figure 2, similarities can be drawn between the mechanisms employed to exploit the host organism for tumorigenesis. The IBP may mediate transitions between latent and lytic viral states, particularly in EBV and KSHV, whereby the IBP’s reliance upon nutrient availability and oxidative stress may indicate a regulatory role for the IBP in the modulation of viral activity [71,97]. Furthermore, since prenylated small GTPases regulate processes such as intracellular trafficking, autophagy, and signal transduction, alterations in IBP flux may potentially influence transcriptional regulators involved in viral lytic switching [63,71,98]. This proposed relationship is however incompletely characterized and requires further investigation.

Beyond its role in intracellular signaling and protein trafficking, IBP-associated lipid remodeling may also influence extracellular vesicle biology [99,100]. Cholesterol-containing membrane domains and prenylated proteins are critical regulators of exosome biogenesis and vesicular transport; thus, it is possible that virally induced changes in lipid metabolism may influence extracellular vesicle tumor microenvironment remodeling [101,102]. Overall, IBP alterations may support extracellular vesicle dynamics via prenylation of small GTPases and cholesterol-dependent membrane reorganization; however, this proposed mechanism is poorly characterized.

Despite promising preclinical findings, the therapeutic intervention of IBP is subject to several limitations. Because GTPases can undergo prenylation switching, isolating a single IBP intermediate may be unrealistic, as compensatory pathways may counteract intended inhibitory actions. This explains why broad IBP inhibition through statins often appears more effective than downstream IBP inhibitors; however, off-target cytotoxicity and compensatory metabolic rewiring introduce complex challenges for achieving therapeutic doses. These limitations suggest that future strategies should focus on combination therapeutic approaches such as simultaneous FTase and GGTase inhibition or synergistic targeting of upstream metabolic pathways with immunotherapies to overcome resistance mechanisms [103,104].

Many studies discussed here showcase the importance of targeting the IBP to slow down pro-oncogenic signaling pathways; however, the limited information regarding the extent of IBP dysregulation in half of the oncoviruses discussed highlights a gap in research that should be addressed to further understanding of how oncoviruses perturb cellular pathways. Establishing more concrete links between the IBP and viral oncogenesis may provide a novel approach to treatment and disease control for many devastating malignancies. As a wide availability of IBP inhibitors exists, namely statins and bisphosphonates, repurposing of these compounds for use as anti-cancer agents may not only provide effective treatments against viral cancers but also allow for the study of poorly understood mechanisms underlying oncogenic viruses.

## Figures and Tables

**Table 1:** Summary of the dysregulated IBP genes and biological consequences associated with the seven oncogenic viruses.

Virus	Viral Family	Genome	Dysregulated IBP Genes	Biological Consequence of Dysregulated IBP	References
Epstein–Barr Virus (EBV)	*Herpesviridae*	DNA (Double-stranded)	*HMGCS1 HMGCR* *SREBP*	IBP activation supports B cell transformation, proliferation and survival. GTPase activation promotes LMP1/LMP2A trafficking. Metabolic dysregulation promotes metastasis and exosome-mediated signaling FTI-277 decreases growth and reduces cellular motility and migration.	[22,23,24,25,26]
Hepatitis B Virus (HBV)	*Hepadnaviridae*	DNA (Double-stranded)	*SREBP2* *HMGCR* 3-hydroxy-3-methylglutarate	Enhanced IBP activity promotes cholesterol accumulation and oncogenic signaling. Increased cholesterol supports viral replication and hepatocarcinogenesis.	[27,28,29,30,31,32,33,34]
Hepatitis C Virus (HCV)	*Flaviviridae*	RNA (Single-stranded)	*HMGCR* *GGPPS*	IBP activity supports HCV viral replication, NS5A complex formation, membrane remodeling and YAP-mediated oncogenic signaling. Geranylgeranylation of host proteins supports viral replication machinery	[35,36,37,38,39,40,41,42]
Human Papillomavirus (HPV)	*Papovaviridae*	DNA (Double-stranded)	N/A	Increased IBP activity supports HPV-associated proliferation, cytoskeletal remodeling and survival via increased GTPase signaling.	[43,44]
Kaposi Sarcoma-associated Herpes Virus (KSHV)	*Herpesviridae*	DNA (Double-stranded)	*HMGSC1*, *HMGCR* *FDFT1*	IBP activity is repressed by viral miRNAs to mediate antiviral responses. Increased cholesterol biosynthesis supports viral infection and latency	[45,46,47,48]
Human T cell lymphotropic virus (HTLV-1)	*Retroviridae*	RNA (Single-stranded)	*FPPS*	Increased abundance of IBP intermediates, particularly GGPP, is critical for HTLV-1-associated ATL survival, proliferation and Rho GTPase activity.	[49,50,51,52,53,54]
Merkel Cell Carcinoma (MCC)	*Polyomavirdae*	DNA (Double-stranded)	*SREBP1/2* Squalene Synthase	Increased IBP flux may support MCC growth, PI3K/AKT signaling and Rho GTPase-mediated motility.	[55,56,57,58]

**Table 2:** Summary of IBP inhibitors investigated as anti-viral agents and their associated impacts on viral infection.

Virus	IBP Inhibitor	In Vitro/In Vivo	Consequences of Viral Infection	Clinical Status	References
Epstein–Barr Virus (EBV)	1. Simvastatin 2. Atorvastatin 3. FTI-277	In vitro	1. Induced apoptosis and inhibited EBV-transformed cell growth. 2. Impaired EBV-driven B-cell growth and survival 3. Disrupted LMP1 exosome loading	Experimental	[22,24,26,63,64,65]
Hepatitis B Virus (HBV)	1. Lovastatin 2. Simvastatin	In vitro	1. Reduced HBsAg secretion 2. Antiviral activity. Combination therapy decreased proliferation and increased apoptosis	Experimental	[27,31,32,33,34]
Hepatitis C Virus (HCV)	1. Atorvastatin 2. Lovastatin 3. GGTI-298 4. MOP compounds	In vitro	1. Reduced YAP signaling 2. Reduced HCV RNA replication and NS5A/FBL2-associated replication machinery. 3. Decreased HCV RNA replication and disrupted NS5A localization 4. Reduced HCV RNA replication	Experimental	[35,36,37,38,39]
Human Papillomavirus (HPV)	1. Atorvastatin, Fluvastatin, Simvastatin 2. 25HC 3. GGTI-298	In vitro	1. Reduced viability and increased apoptosis/necrosis in HPV positive cell lines 2. Impaired HPV-associated cytoskeletal remodeling 3. Activation of key host proteins LIMK1/Cofflin was prevented and tumorigenic cytoskeletal dynamics perturbed.	Experimental	[43,44,66,67,68]
Kaposi Sarcoma-associated Herpes Virus (KSHV)	25HC	In vitro	Decreased viral gene expression and enhanced antiviral interferon responses.	Experimental	[69]
Human T cell lymphotropic virus (HTLV-1)	1. Lovastatin & Simvastatin 2. Incadronate 3. GGTI-298	In vitro	1. Decreased ATL cell viability 2. Induced apoptosis and increased unprenylated Rap1A levels in HTLV-1 positive cells.	Experimental Preclinical	[49,50,51]
Merkel Cell Carcinoma (MCC)	Selinexor	In vitro	Decreased MCC cell growth and indirect suppression of IBP gene expression.	Experimental	[56]

**Table 3:** MicroRNA-mediated regulation of the IBP in the seven oncogenic viruses.

Virus	MicroRNA	IBP Target	Biological Consequence	References
Epstein–Barr Virus (EBV)	No established IBP-associated microRNAs	N/A	N/A	N/A
Hepatitis B Virus (HBV)	miR-21 miR-27	HMGCR Cholesterol pathways	Regulation of cholesterol production and IBP activity.	[19,76]
Hepatitis C Virus (HCV)	miR-21 miR-27 miR-122 miR-185-5p	HMGCR SREBP Cholesterol metabolism and HCV RNA regulation	High miRNA expression modulates IBP activity. miR-27 represses HMGCR.	[76,79,80,81]
Human Papillomavirus (HPV)	No established IBP-associated microRNAs	N/A	N/A	N/A
Kaposi Sarcoma-associated Herpes Virus (KSHV)	Viral KSHV miRNA	HMGCS1, HMGCR, FDFTI, CH25H	miRNAs repress IBP-associated genes, reduce 25HC antiviral responses and promote viral latency and survival	[45,46]
Human T cell lymphotropic virus (HTLV-1)	No established IBP-associated microRNAs	N/A	N/A	N/A
Merkel Cell Carcinoma (MCC)	No established IBP-associated microRNAs	N/A	N/A	N/A

## Data Availability

No new data were created or analyzed in this study.

## Abbreviations

The following abbreviations are used in this manuscript:

IBP	Isoprenoid Biosynthesis pathway
FPP	Farnesyl pyrophosphate
GGPP	Geranylgeranyl pyrophosphate
HMGCR	HMG-CoA reductase
EBV	Epstein–Barr virus
HBV	Hepatitis B virus
HCV	Hepatitis C virus
HPV	Human Papillomavirus
KSHV	Kaposi sarcoma-associated herpes virus
HTLV	Human T cell lymphotropic virus
MCC	Merkel cell carcinoma
IPP	Isopentenyl pyrophosphate
DMAPP	Dimethylallyl pyrophosphate
GPP	Geranyl pyrophosphate
FPPS	FPP synthase
GGPPS	GGPP synthase
FTase	Farnesyl transferase
GGTase	Geranylgeranyl transferase
ER	Endoplasmic reticulum
SREBP	Sterol regulatory element binding proteins
SCAP	SREBP cleavage-activating protein
HMGCS	HMG-CoA synthase
LMP	Latent membrane protein
LFA	Leukocyte function antigen
FTI	Farnesyl transferase inhibitor
GGTI	Geranylgeranyl transferase inhibitor
HBsAg	HBV-associated viral antigen
CHB	Chronic hepatitis B infection
miR	MicroRNA
YAP	Yes-associated protein
MOP	Monascus orange pigment
25HC	25-hydroxycholesterol
CDP	Bacterial cyclodipeptides
ATL	Adult T cell leukemia-lymphoma
MCPyV	Merkel cell polyomavirus
